# Advances in Key Genetic Elements and Strategies for High-Yield Heterologous Protein Expression in *Komagataella phaffii*

**DOI:** 10.3390/jof12080612

**Published:** 2026-08-15

**Authors:** Ruyue Han, Ruizheng Hu, Anran Liu, Xinya Yu, Dong Liu, Hengwei Zhang, Hailing Zhang

**Affiliations:** College of Life Sciences, Yantai University, 30 Qingquan Road, Yantai 264005, China; ruyue_han@163.com (R.H.); ruizheng37@s.ytu.edu.cn (R.H.); lar2317837959@163.com (A.L.); 15553792606@163.com (X.Y.); liudong2021@ytu.edu.cn (D.L.)

**Keywords:** *Komagataella phaffii* (*K. phaffii*), heterologous protein expression, promoter engineering, signal peptide engineering, protein secretion, synthetic biology

## Abstract

*Komagataella phaffii* has emerged as an important eukaryotic microbial cell factory for the production of industrial enzymes, biopharmaceuticals, and food-related proteins owing to its rapid growth, capacity for eukaryotic post-translational modifications, and suitability for high-cell-density fermentation. However, efficient heterologous protein production remains constrained by limitations in transcriptional regulation, protein secretion, and endoplasmic reticulum (ER) protein-folding capacity. This review summarizes recent advances in engineering key expression elements underlying heterologous protein production in *K. phaffii*, with particular emphasis on promoter architecture redesign (e.g., upstream activating sequence (UAS) duplication and core promoter mutagenesis), signal peptide replacement and sequence engineering, molecular chaperone co-expression, and quantitative regulation of the unfolded protein response (UPR). Rather than focusing on individual expression modules, this review highlights a systems-level engineering perspective that integrates regulatory elements, intracellular processing, and secretory pathway optimization, illustrating the ongoing transition from empirical modification of individual expression elements to systems-level engineering of the secretory pathway. Recent advances in synthetic biology and artificial intelligence (AI)-assisted approaches are further accelerating the development of predictive and precision engineering approaches for *K. phaffii*. Together, these advances provide a foundation for the rational design of next-generation *K. phaffii* cell factories for the sustainable production of structurally complex and high-value recombinant proteins.

## 1. Introduction

As one of the most widely used eukaryotic expression hosts, *Komagataella phaffii* (formerly *Pichia pastoris)* has been granted Generally Recognized as Safe (GRAS) status by the U.S. Food and Drug Administration (FDA) and has been employed for the production of over 5000 recombinant proteins [1]. Owing to its genetic stability, efficient post-translational modification capability, and suitability for high-cell-density fermentation, *K. phaffii* has become a leading microbial platform for heterologous protein production [2]. Under optimized fermentation and strain engineering conditions, recombinant protein production in *K. phaffii* can reach gram-per-liter to tens-of-grams-per-liter levels, although the achievable titers remain highly dependent on the properties of individual proteins, strain background, and cultivation processes [3,4,5]. These high productivities highlight the substantial production capacity of this yeast platform while also revealing the importance of balancing transcriptional output, protein folding, and secretion capacity [6,7]. Compared with both prokaryotic and mammalian expression hosts, *K. phaffii* provides an attractive balance between low production cost, rapid cultivation, scalability, and eukaryotic protein processing, making it particularly suitable for the large-scale production of industrial enzymes, biopharmaceuticals, vaccine antigens, and other functional proteins [8,9,10,11,12,13]. Although many engineering strategies currently employed in *K. phaffii* were originally established in *Saccharomyces cerevisiae*, the two yeasts exhibit both conserved molecular mechanisms and important physiological differences. As closely related budding yeasts, they share highly conserved transcriptional machinery, ER protein-folding networks, unfolded protein response (UPR), ER-associated degradation (ERAD), and vesicle trafficking pathways [14,15,16]. Consequently, numerous promoter engineering, signal peptide optimization, and molecular chaperone engineering strategies developed in *S. cerevisiae* have provided valuable guidance for engineering *K. phaffii*. However, *K. phaffii* possesses unique physiological characteristics, including methanol utilization, superior high-cell-density fermentation capability, and lower endogenous protein secretion, making it particularly advantageous for large-scale recombinant protein production [17]. As the applications of recombinant proteins continue to expand across medicine, food, agriculture, materials science, and industrial biotechnology, increasing demands are being placed on expression platforms with greater productivity, robustness, and manufacturing efficiency [8].

Despite these advantages, efficient heterologous protein production in *K. phaffii* remains challenging because recombinant protein biosynthesis depends on the precise coordination of multiple cellular processes. These processes are governed by a series of interconnected expression elements that regulate transcription, protein translocation, folding, post-translational processing, and secretion. Among them, promoters determine the transcriptional output of heterologous genes [18]. Signal peptides direct nascent polypeptides into the secretory pathway and largely influence secretion efficiency, whereas molecular chaperones facilitate protein folding, maintain protein homeostasis, and improve recombinant protein stability and secretion [19]. Consequently, engineering these expression elements has become a central strategy for enhancing heterologous protein production. Recent advances in synthetic biology have facilitated promoter architecture redesign through UAS duplication and transcription factor-binding site mutagenesis, signal peptide replacement and α-factor sequence engineering, co-expression of ER-resident molecular chaperones, vector engineering, multicopy genome integration, and CRISPR-based genome editing [16]. These approaches directly target the major bottlenecks in transcription, protein translocation, endoplasmic reticulum (ER) folding capacity, and secretory pathway efficiency. Together, these strategies provide complementary solutions for improving recombinant protein maturation and extracellular production [20]. Although substantial progress has been achieved, these engineering strategies are often investigated independently, and a comprehensive understanding of their coordinated regulation and synergistic effects remains limited.

Importantly, the engineering strategies required for heterologous protein production in *K. phaffii* depend not only on the expression system but also on the structural characteristics of the target protein. Large multidomain proteins often require coordinated optimization of protein translocation, folding, and secretion, whereas disulfide-rich proteins place greater demands on the ER oxidative folding machinery [8,9,21]. Likewise, membrane proteins frequently benefit from optimized co-translational targeting and membrane insertion strategies, while multimeric proteins require efficient assembly and intracellular trafficking [18]. Accordingly, this review discusses recent advances in promoter engineering, signal peptide engineering, molecular chaperone engineering, and other key expression elements from the perspective of their application to different classes of recombinant proteins whenever appropriate [22]. It should be noted that the engineering strategies discussed in this review are primarily focused on the production of secreted soluble recombinant proteins, representing the most widely investigated application of the *K. phaffii* expression platform. For this class of proteins, promoter selection, signal peptide optimization, ER folding capacity, and secretory pathway engineering represent major determinants of production efficiency. However, recombinant proteins with distinct structural characteristics require additional specialized considerations. For example, disulfide-rich proteins often require enhanced oxidative folding capacity through optimization of protein disulfide isomerase (PDI), endoplasmic reticulum oxidoreductases, and unfolded protein response (UPR) regulation [23,24,25]. In contrast, membrane proteins present unique challenges associated with hydrophobic transmembrane domains, membrane insertion efficiency, and potential toxicity to host cells, requiring optimization of co-translational targeting pathways, membrane-associated factors, and expression levels [7,25]. Similarly, large multidomain or oligomeric proteins may require coordinated regulation of folding, assembly, and intracellular trafficking [21,26]. Although these specialized strategies are beyond the primary scope of this review, their importance highlights the necessity of developing protein-specific engineering approaches rather than applying universal optimization strategies to all recombinant products.

**Figure 1:**
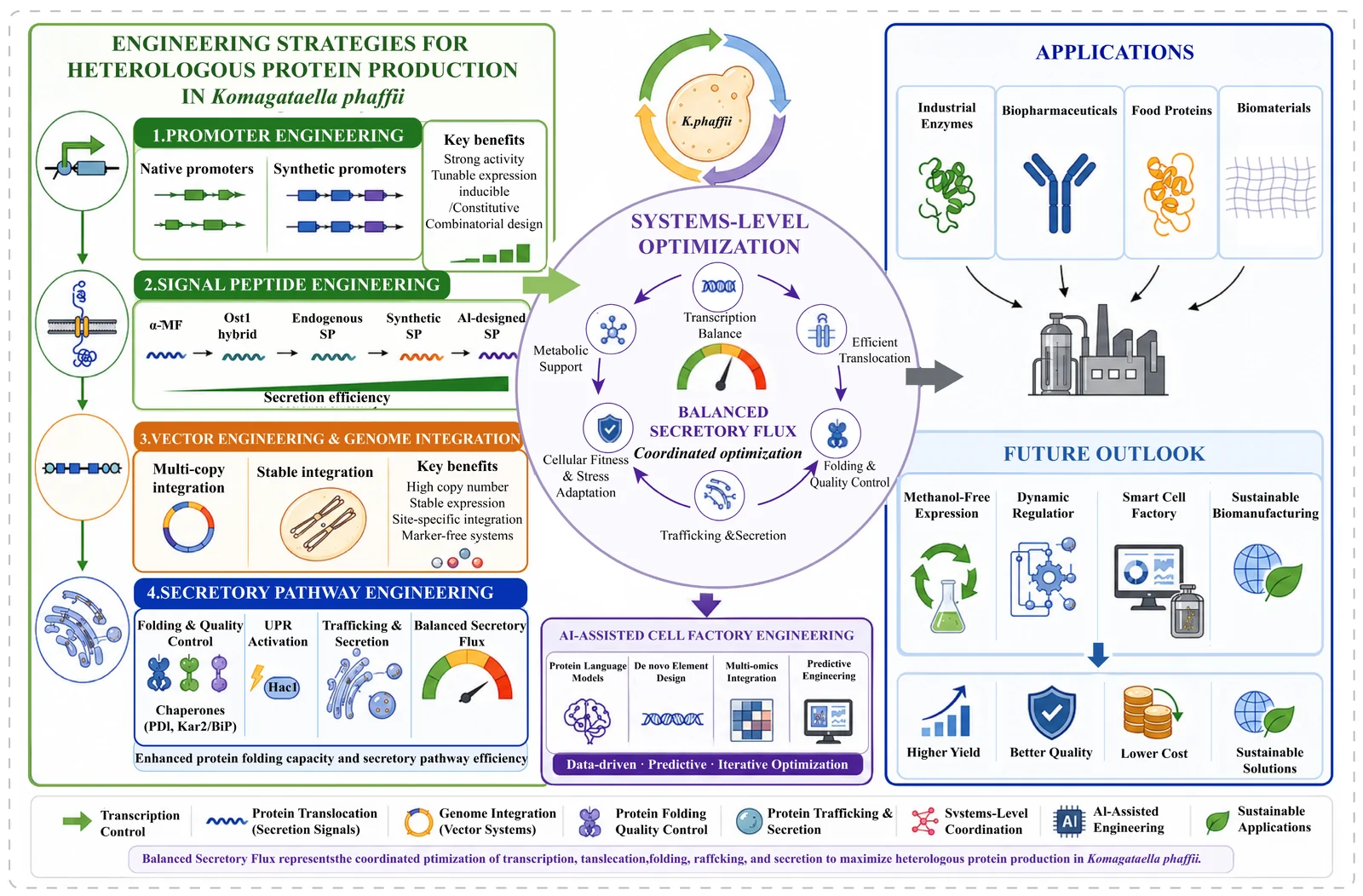
Schematic overview of engineering strategies for enhancing heterologous protein production in *K. phaffii*. Promoter engineering, signal peptide engineering, vector engineering and genome integration, and secretory pathway engineering are integrated through systems-level optimization to establish balanced secretory flux and maximize recombinant protein production. Emerging AI-assisted engineering strategies support the development of next-generation smart cell factories for sustainable biomanufacturing and diverse industrial applications.

This review provides a comprehensive overview of recent advances in the key expression elements governing heterologous protein production in *K. phaffii*. Because the physiological constraints and engineering requirements differ substantially between conventional methanol-induced expression systems (primarily PAOX1-based) and alternative transcriptional control systems, including constitutive, methanol-free, and dynamically regulated promoters, these engineering strategies are discussed within the context of their corresponding transcriptional backgrounds whenever applicable. As illustrated in Figure 1, promoter engineering, signal peptide engineering, vector engineering and genome integration, and secretory pathway engineering are integrated through systems-level optimization to achieve balanced secretory flux and maximize recombinant protein production. The framework further highlights emerging research directions, including AI-assisted cell factory engineering and sustainable biomanufacturing. Rather than simply accelerating strain development, these approaches are expected to address current challenges in identifying optimal combinations of promoters, signal peptides, and folding modules, thereby reducing empirical trial-and-error during strain construction and facilitating more efficient engineering of protein-specific expression systems [10]. Particular emphasis is placed on synthetic biology-enabled engineering strategies, including promoter architecture redesign, signal peptide replacement and sequence engineering, molecular chaperone co-expression and ER folding network engineering, vector engineering and genome integration, and CRISPR-based genome editing, with a focus on their coordinated regulation and synergistic effects on recombinant protein expression and secretion. The review first discusses the regulatory characteristics of native and engineered promoters, followed by recent advances in signal peptide discovery and optimization, vector engineering and genome integration, molecular chaperone engineering for improving protein folding and secretion, and finally the remaining challenges and future perspectives. Collectively, these advances provide important insights into the rational design of next-generation *K. phaffii* cell factories for industrial and biotechnological applications.

## 2. Promoters: Key Regulatory Elements for Heterologous Protein Expression

Promoters are the primary cis-regulatory elements governing transcription initiation and therefore represent one of the most important determinants of heterologous protein expression in *K. phaffii*. By controlling the recruitment of RNA polymerase II and gene expression factors, promoters directly influence transcriptional output and ultimately determine recombinant protein productivity [4]. Consequently, promoter selection and engineering have become fundamental strategies for optimizing *K. phaffii* expression systems.

**Figure 2:**
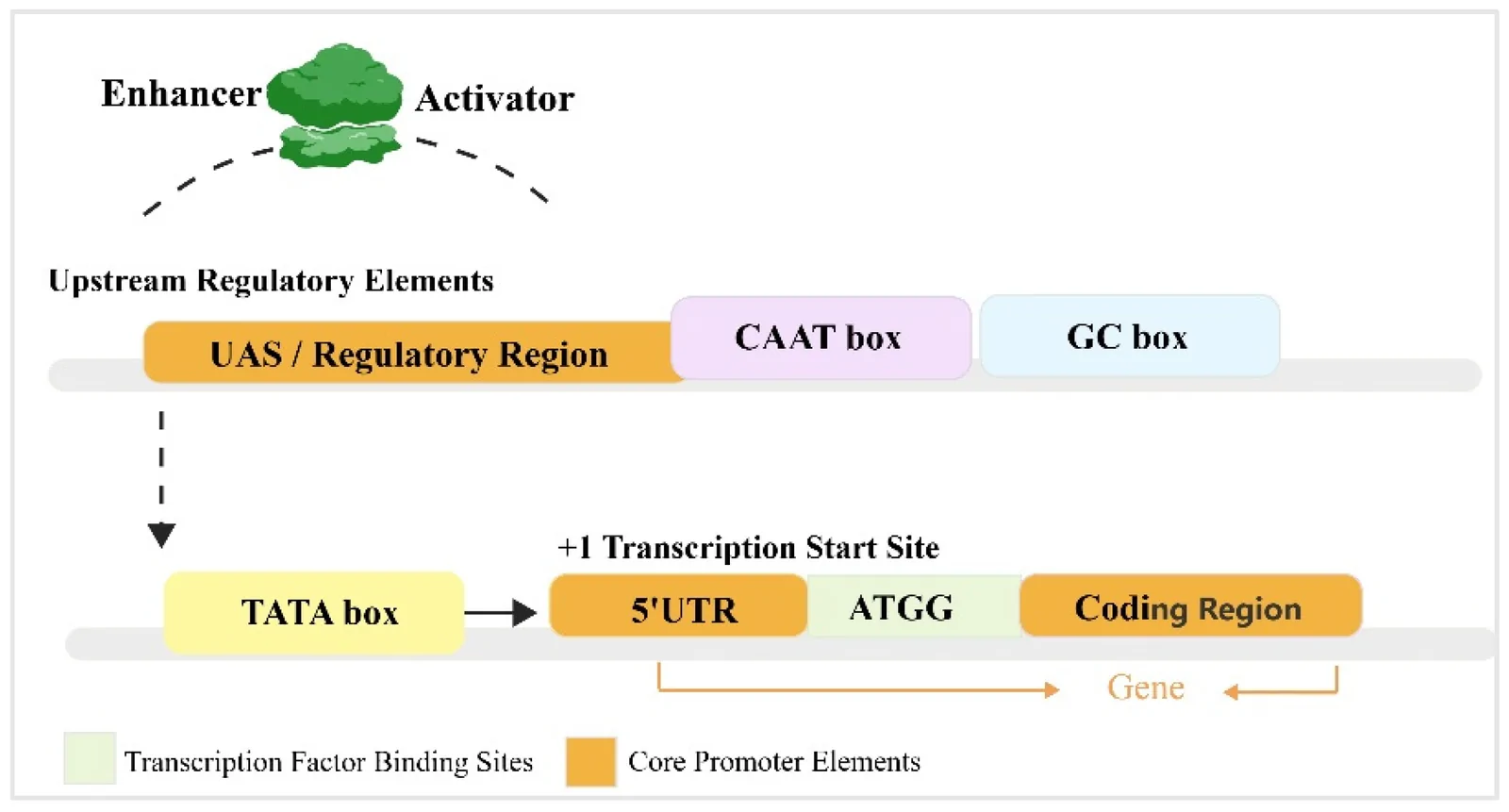
Modular architecture of a typical *K. phaffii* promoter.The schematic illustrates the organization of regulatory and core promoter elements involved in transcriptional regulation. Dashed arrows indicate regulatory interactions between elements, whereas solid arrows indicate the directional relationship or sequential arrangement of genetic elements.

As illustrated in Figure 2, the modular architecture of *K. phaffii* promoters provides multiple engineering targets for regulating gene transcription. Modifications of the core promoter primarily affect transcription initiation, whereas modification of upstream cis-regulatory elements enables dynamic regulation of promoter strength and environmental responsiveness [7]. This modular organization forms the structural basis for modern promoter engineering.The promoter consists of an upstream regulatory region containing upstream activating sequences (UASs), CAAT and GC boxes, and a core promoter comprising the TATA box, transcription start site (TSS), and 5′ untranslated region (5′-UTR) [27,28]. Together, these modules regulate transcription initiation and provide the structural framework for promoter engineering.

Recent advances in synthetic biology, systems biology, and computational biology have fundamentally transformed promoter engineering. Instead of relying solely on empirical screening of naturally occurring promoters, researchers now employ promoter libraries, cis-regulatory element engineering, transcription factor overexpression and transcription factor-binding site (TFBS) engineering, hybrid promoter construction, and artificial intelligence-assisted sequence design to generate promoters with tunable and predictable transcriptional activities. Synthetic core promoters, hybrid promoter libraries, and transcriptional engineering have substantially expanded the regulatory flexibility of *K. phaffii* promoters, providing diverse tools for fine-tuning gene expression under different production requirements [27,28,29,30]. More recently, artificial intelligence and deep-learning approaches have enabled the rational design of synthetic promoters with predictable activities, further accelerating promoter architecture redesign from empirical promoter screening toward data-driven design [31,32]. These developments reflect a clear transition from empirical promoter screening toward predictive and programmable transcriptional regulation, thereby facilitating rational design. Transcriptional engineering contributes to the coordinated regulation of transcriptional output and secretory capacity.

Rather than pursuing the strongest possible promoter, current engineering strategies increasingly focus on matching transcriptional output with the protein-folding, secretion, and metabolic capacities of the host. Excessively strong transcription frequently overloads the endoplasmic reticulum and secretory pathway, resulting in protein misfolding, intracellular accumulation, and metabolic burden [25]. Consequently, promoter engineering is evolving from maximizing transcriptional activity to balancing transcriptional output with folding and secretion capacity through dynamic and context-dependent regulation.

Based on their regulatory mechanisms, promoters in *K. phaffii* can be broadly classified into native promoters and engineered promoters. The following sections discuss these promoter categories from the perspectives of regulatory mechanisms, engineering strategies, and industrial applicability, with particular emphasis on how different promoter engineering strategies can be tailored to balance transcriptional activity with the protein-processing capacity and physiological state of the host.

### 2.1. Native Inducible Promoters

Inducible promoters represent the most extensively used transcriptional regulatory elements in *K. phaffii* cell factories because they enable temporal separation of cell growth and recombinant protein production [7,10]. In recent years, their engineering has evolved from conventional methanol-dependent induction toward methanol-free expression systems [33], dynamic regulatory strategies [34], and data-driven promoter design [31]. Among the currently available inducible promoters, the alcohol oxidase 1 promoter (PAOX1) remains the benchmark for heterologous protein production owing to its exceptionally high transcriptional activity. Under fully induced conditions, proteins expressed under the control of PAOX1 may account for more than 30% of the total cellular protein [7,29]. At the fermentation scale, PAOX1-driven expression systems have enabled recombinant protein titers ranging from several grams per liter to above 10 g L^−1^ depending on the target protein, strain background, and downstream secretion capacity [3,8]. However, these high expression levels are frequently limited by bottlenecks in protein folding, ER processing, and secretion efficiency [6,35].

PAOX1 exhibits a tightly regulated induction mechanism in response to carbon source availability [29]. Transcription is repressed in the presence of preferred carbon sources such as glucose and glycerol but is strongly induced upon methanol induction through transcription factors including MXR1p and MIT1p [7,36]. This tightly controlled switch-like expression pattern enables efficient separation of biomass accumulation and recombinant protein production, making PAOX1 the promoter of choice for industrial-scale fermentation [8].

Despite its remarkable transcriptional strength, PAOX1-driven expression often exceeds the protein-folding and secretory capacities of the host cell, thereby creating bottlenecks in protein maturation, translocation, and secretion [6,8,18]. Consequently, recent engineering efforts have shifted from maximizing transcriptional output toward balancing transcription with downstream protein-processing capacity. Representative engineering interventions include dynamic optimization of methanol feeding profiles to reduce ER folding stress during membrane protein production [18], co-expression of prolyl 4-hydroxylase (P4H) to increase proline hydroxylation efficiency during collagen biosynthesis, and modulation of PAOX1 activity through transcription factor engineering of MXR1 and MIT1 [37]. These targeted modifications improve protein maturation rather than merely increasing transcription. Collectively, these studies demonstrate that improving recombinant protein yield increasingly depends on coordinated regulation of transcription, protein folding, and secretion rather than promoter strength alone.

To overcome the inherent limitations of methanol induction, including flammability, volatility, and methanol-associated metabolic toxicity, increasing efforts have been devoted to developing alternative inducible promoters [4,33,38]. These promoters can be broadly classified into metabolite-inducible and carbon source-responsive promoters. Among them, the formaldehyde dehydrogenase promoter (PFLD1) is the most extensively studied metabolite-inducible promoter [29]. It responds to both methanol and methylamine and exhibits transcriptional activity comparable to that of PAOX1 under methylamine induction, while reducing the operational risks associated with methanol handling and providing greater flexibility for industrial fermentation [39].

Another representative promoter is the isocitrate lyase promoter (PICL1), a carbon source-responsive derepressible promoter regulated by endogenous carbon metabolism [40,41]. Although its maximal transcriptional activity is lower than that of PAOX1, PICL1 is particularly suitable for dynamic pathway regulation and stage-specific expression of auxiliary genes because of its moderate and growth-associated expression profile. In addition, the dihydroxyacetone synthase promoters (PDAS1 and PDAS2) exhibit transcriptional strengths comparable to that of PAOX1 while possessing distinct promoter sequences [29,42]. Their incorporation into multigene expression systems effectively reduces homologous recombination caused by repeated PAOX1 sequences, thereby improving the genetic stability of multicopy integration strains without compromising transcriptional output [20,43].

Accumulating evidence indicates that alternative inducible promoters are not intended to replace PAOX1 but rather to complement its regulatory limitations. Their distinct induction mechanisms and transcriptional characteristics provide greater flexibility for multigene expression, dynamic metabolic regulation, and safer industrial fermentation. Increasingly, promoter selection is guided by specific production objectives rather than simply increasing transcriptional output.

Overall, inducible promoters remain the preferred choice for high-level recombinant protein production because they combine strong transcriptional activity with precise temporal regulation of gene expression. However, recent advances indicate that simply maximizing promoter strength does not necessarily improve protein yield. Instead, promoter architecture redesign is shifting toward coordinated regulation of transcription, protein folding, secretion, and cellular metabolism. Most engineering strategies developed for PAOX1-based expression systems have been optimized under methanol induction and are therefore closely associated with methanol metabolism, redox balance, and induction kinetics. Consequently, conclusions derived from PAOX1-driven production cannot always be directly extrapolated to constitutive or methanol-free expression systems, where transcriptional regulation, metabolic burden, and protein secretion constraints differ substantially. Accordingly, the development of methanol-free, dynamically regulated, AI-assisted, and programmable promoters [31] is expected to further improve the biosafety, process flexibility, and industrial applicability of *K. phaffii* expression systems.

### 2.2. Native Constitutive Promoters

Unlike inducible promoters, constitutive promoters continuously drive gene expression without requiring external inducers, thereby simplifying fermentation processes and facilitating industrial scale-up [2,8,39]. Because their activities are closely coupled with cellular growth and metabolism, constitutive promoters are particularly suitable for continuous protein production, metabolic engineering, and multigene pathway regulation [35]. Most constitutive promoters are derived from housekeeping genes involved in central carbon metabolism or the translational machinery, enabling recombinant protein synthesis to proceed concurrently with biomass accumulation [27]. Among the available constitutive promoters, the glyceraldehyde-3-phosphate dehydrogenase promoter (PGAP), translation elongation factor 1 promoter (PTEF1), and phosphoglycerate kinase promoter (PPGK1) are the most widely employed because of their robust activities and broad applicability in *K. phaffii*.

Among constitutive promoters, PGAP is the most extensively used owing to its strong, growth-associated transcriptional activity [27,44], which is closely linked to glycolytic carbon flux. Unlike PAOX1, PGAP provides sustained transcription throughout the exponential growth phase, making it particularly suitable for continuous recombinant protein production [45]. Representative studies have demonstrated that PGAP has been reported to outperform PAOX1 for several recombinant proteins in both secreted and intracellular protein expression. For example, enhanced production of recombinant human α1-antitrypsin was attributed to reduced endoplasmic reticulum folding stress under continuous transcription [46], whereas improved intracellular phytase production reflected better coordination between protein synthesis and host metabolism during high-cell-density cultivation [47]. Collectively, these studies indicate that the advantages of PGAP arise not only from its transcriptional strength but also from its ability to synchronize protein synthesis with cellular metabolic activity, thereby reducing metabolic burden and facilitating efficient protein maturation [44].

In addition to PGAP, other constitutive promoters provide complementary regulatory characteristics for specific engineering objectives. PTEF1, derived from the translation elongation factor gene, is regulated independently of glycolytic metabolism and is therefore frequently combined with PGAP to balance transcriptional resources in multigene expression systems [44,45]. By rationally combining PGAP, PTEF1, and their truncated derivatives, Huang et al. [45] successfully optimized heterologous leech hyaluronidase production, demonstrating that coordinated promoter selection is more effective than relying on a single strong promoter for pathway optimization.

Compared with PGAP and PTEF1, PPGK1 generally exhibits moderate transcriptional activity and is particularly suitable for auxiliary genes whose overexpression may impose unnecessary metabolic burden [29]. Consequently, PPGK1 has been widely applied to regulate molecular chaperones, transporter proteins, and other supporting genes in synthetic metabolic pathways [39]. In addition, heterologous constitutive promoters, such as PTPI from *Kluyveromyces marxianus* and PPMA from *Ogataea polymorpha*, have attracted increasing attention because their low sequence homology with the *K. phaffii* genome minimizes homologous recombination during multicopy integration, thereby improving the genetic stability of engineered strains while maintaining reliable transcriptional activity under industrial fermentation conditions [39].

Taken together, constitutive promoters exhibit distinct regulatory characteristics and should therefore be selected according to specific engineering objectives rather than simply selecting the strongest available promoter. Unlike methanol-induced PAOX1 systems, constitutive promoters continuously drive recombinant protein synthesis throughout cell growth. As a result, protein-folding stress, secretion capacity, and cellular metabolism are regulated under fundamentally different physiological conditions. PGAP is generally preferred for high-level recombinant protein production because of its robust growth-associated expression, whereas PTEF1 is particularly suitable for balancing multigene expression owing to its distinct regulatory mechanism. In contrast, promoters with moderate activities, such as PPGK1, provide greater flexibility for optimizing pathway flux and reducing metabolic burden, while heterologous promoters further improve strain stability by minimizing homologous recombination during genome integration. Consequently, optimization strategies developed for constitutive expression systems frequently emphasize long-term proteostasis, metabolic balance, and sustained secretion rather than induction efficiency.

These advances demonstrate that constitutive promoter engineering has evolved from identifying stronger native promoters toward rational allocation of transcriptional resources. Rather than maximizing expression of individual genes, current strategies increasingly employ promoters with different strengths and regulatory mechanisms to coordinate pathway flux, reduce metabolic burden, and improve long-term production stability. This transition reflects a broader shift from transcription-centered optimization toward systems-level regulation of recombinant protein production. Nevertheless, the regulatory range of native constitutive promoters remains inherently limited. Consequently, rational engineering of promoter architectures has become increasingly important for achieving programmable transcriptional regulation through promoter engineering, as discussed in the following section.

### 2.3. Promoter Engineering

**Figure 3:**
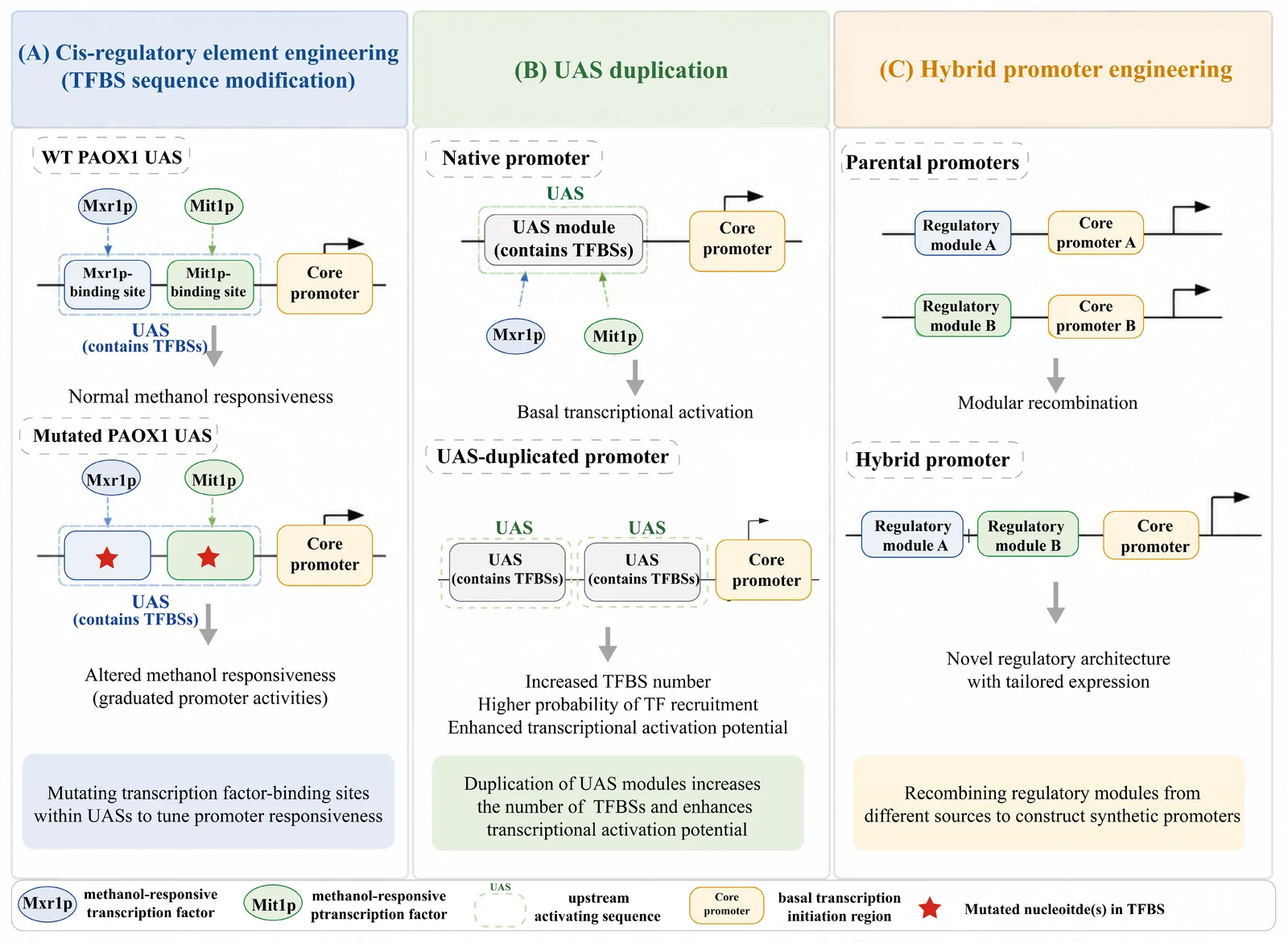
Mechanistic strategies for promoter engineering in *K. phaffii*. (**A**) Modification of transcription factor-binding sites (TFBSs) within upstream activating sequences (UASs) tunes promoter responsiveness. (**B**) Duplication of UAS modules increases the number of TFBSs and enhances transcriptional activation potential. (**C**) Hybrid promoter engineering combines regulatory modules from different promoters to construct synthetic promoters with tailored transcriptional activities [28,48].

Although native promoters provide a robust transcriptional foundation for heterologous protein production, their regulatory characteristics are inherently constrained by naturally evolved promoter architectures [7,8]. Consequently, rational core promoter replacement has become a central strategy for expanding the regulatory flexibility of *K. phaffii* expression systems [10]. Rather than simply replacing native promoters, current engineering approaches increasingly focus on redesigning promoter architectures [31,32] to achieve programmable and predictable transcriptional regulation. Because the fundamental transcriptional machinery is highly conserved between *S. cerevisiae* and *K. phaffii*, many promoter engineering strategies, including cis-regulatory element engineering, transcription factor engineering, and synthetic promoter design, were initially developed in *S. cerevisiae* before being adapted to the methanol-regulated transcriptional network of *K. phaffii*. As illustrated in Figure 3, promoter architecture redesign primarily targets two functional modules: upstream cis-regulatory elements [28,48], which determine transcription factor recruitment and regulatory responsiveness, and the core promoter region, which governs transcription initiation. Advances in synthetic biology have further extended these strategies toward modular promoter assembly [10,31,32], transcription factor engineering, and intelligent promoter design, enabling increasingly precise control of heterologous gene expression.Representative promoter engineering approaches, including cis-regulatory element modification, core promoter optimization, hybrid promoter construction, and transcription factor-based regulation, together with their applications in *K. phaffii* expression systems, are summarized in Table 1.

**Table 1:** Representative promoter engineering strategies and their applications for enhancing heterologous protein expression in *K. phaffii*.

Strategy	Promoter	Target Protein	Relative Improvement	Final Titer	Reference
Cis-module engineering	PAOX1/PS2	3-HP	Promoter activity increased (~2-fold)	22 g/L	Chen et al., 2025 [43]
TF engineering((Mxr1 positive feedback)	PAOX1/PAOX2	scFv	PAOX2 transcription increased (2.0–2.7-fold)	NR	Chang et al., 2018 [36]
TF network engineering	PAOX1	*Rhizopus oryzae* lipase (ROL)	~5-fold higher lipase activity	NR	Cámara et al., 2019 [49]
Core promoter mutagenesis	PGAP	GFP	0.35- to 3.10-fold of the wild-type P_GAP expression strength	NR	Ata et al., 2017 [44]
Hybrid promoter library construction	PGAP/Phy47	GFP	Promoter activity enhanced (~2.9-fold)	NR	Lai et al., 2023 [30]
UPR feedback	PTEF1	GFP	ER stress-responsive promoter activity increased (~3–4-fold)	NR	Kullawong et al., 2018 [50]
Metabolic engineering	PAOX1	Industrial enzymes	Protein expression reached 82–95% of the PAOX1 methanol-induced level without methanol	NR	Bustos et al., 2024 [51]
Core replacement	PsynV-5/P068	α-Amylase	Promoter activity enhanced (2.6~4.5-fold)	NR	Liu et al., 2025 [52]
Transcription factor engineering	PGAP	Xylanase	Xylanase expression increased (~2.43-fold)	NR	Lin et al., 2024 [27]
Truncation	Endogenous promoters	HRP	Tunable promoter variants with different transcriptional strengths	NR	Vogl et al., 2016 [29]
de novo synthetic core promoters	PTPI/PPMA	GFP	Stable constitutive promoter activity in orthogonal expression system	NR	Portela et al., 2017 [28]
Bidirectional promoters	HHT1/HHX1	GFP	Coordinated co-expression of multiple genes	NR	Vogl et al., 2018 [42]

Abbreviations: NR, not reported. Absolute protein titers (g L^−1^ or mg L^−1^) are provided whenever available in the original publications. For promoter engineering studies, the primary evaluation metrics were frequently promoter activity, transcription level, or reporter fluorescence rather than volumetric recombinant protein production. Therefore, only relative promoter activities are reported for studies in which absolute protein titers were unavailable.

#### 2.3.1. Engineering of Cis-Regulatory Elements

Cis-regulatory engineering represents the foundation of promoter optimization in *K. phaffii* by modifying upstream activating sequences (UASs) and core promoter elements to tune transcriptional output [28,48]. Whereas UAS engineering primarily reshapes transcription factor recruitment and environmental responsiveness, core promoter engineering regulates basal transcription initiation efficiency [29].

Representative studies have demonstrated these two complementary mechanisms. Vogl et al. [29,42] engineered PAOX1 by selectively mutating MXR1- and MIT1-binding sites within the upstream activating sequences (UASs), thereby generating promoter variants exhibiting graded methanol responsiveness rather than simply stronger transcription. In contrast, Ata et al. performed saturation mutagenesis of nucleotides surrounding the TATA box within the PGAP core promoter, generating variants with basal transcription activities ranging from 0.35- to 3.10-fold relative to the native promoter [28,44].

Accumulating evidence suggests that cis-regulatory engineering shifts promoter design from empirical promoter optimization toward modular and quantitative control of transcriptional output, enabling predictable expression tuning across diverse production conditions. Across reported studies, the highest recombinant protein titers achieved in *K. phaffii* generally fall within the tens-of-grams-per-liter range under optimized fermentation conditions. However, such high yields are typically achieved through combined optimization of promoters, secretion signals, folding capacity, and fermentation processes rather than through enhancement of a single genetic element.

#### 2.3.2. Hybrid Promoter Engineering

While cis-element engineering optimizes existing promoter architectures, hybrid promoter engineering creates entirely new regulatory combinations through modular recombination. Hybrid promoter engineering extends cis-regulatory design by recombining functional promoter modules to generate synthetic architectures with enhanced or customized transcriptional behavior [30,42]. By integrating heterologous or endogenous regulatory elements, this strategy enables simultaneous improvement of transcriptional strength and regulatory adaptability [28,30]. For example, Lai et al. [30] generated the synthetic promoter phy47 by fusing a synthetic upstream activating sequence to the native PGAP core promoter, thereby increasing transcriptional activity without altering downstream coding sequences. Similarly, transcriptome-guided reconstruction of active regulatory elements enabled the rational redesign of PGAP, leading to improved xylanase production under industrial conditions [27].

Compared with single-element modification, hybrid promoter engineering provides greater design flexibility [32] and expands the accessible transcriptional space. However, promoter performance remains context-dependent due to complex interactions among cis-elements, transcription factors, and chromatin structure, often requiring empirical optimization.

#### 2.3.3. Engineering of Trans-Acting Factors

Whereas cis- and hybrid promoter engineering operate at the DNA sequence level, regulation through MIT1/MXR1 overexpression and TF-binding-site modification at the network level by modifying the activity of shared regulatory proteins [36,49]. This strategy enables coordinated control of multiple promoters by modulating transcription factor abundance, activity, or interaction networks [36].

Overexpression of *MIT1* [36] increased PAOX1 activation by enhancing binding of the transcription factor to AOX1 upstream activating sequences, whereas co-engineering of *MXR1* [36,49] and *MIT1* simultaneously altered multiple methanol-responsive regulatory pathways.

Unlike promoter-centric strategies, modulation of MIT1 and MXR1 enables system-wide reprogramming of transcriptional networks. However, due to the pleiotropic roles of transcription factors in metabolism and stress responses [10], achieving predictable and stable expression remains challenging.

#### 2.3.4. Intelligent Promoter Design

Building upon promoter architecture and transcription factor engineering, recent advances have shifted the field toward intelligent and adaptive regulatory systems capable of autonomously responding to intracellular physiological states and environmental cues [10,31]. These strategies integrate synthetic regulatory circuits, multigene coordination, and machine learning-based sequence design to achieve dynamic transcriptional control.

For instance, incorporation of unfolded protein response (UPR) elements [50] into synthetic promoters enables feedback regulation of transcription in response to ER stress, thereby balancing protein synthesis with folding capacity. In parallel, bidirectional promoter [42] systems constructed from PGAP and PAOX1 variants carrying TFBS modifications allow compact and coordinated multigene expression while minimizing homologous recombination. Moreover, machine learning approaches [31] have been applied to identify sequence features associated with promoter activity and to guide de novo promoter design, reducing reliance on empirical screening [32].

Recent studies suggest that intelligent promoter engineering represents a transition from static regulation toward adaptive gene expression systems. Rather than replacing experimental optimization, machine-learning models are primarily used to prioritize promoter variants, predict transcriptional strength, and identify sequence features associated with promoter performance before experimental validation [31,32]. These approaches are particularly valuable for reducing the size of experimental libraries and accelerating the identification of promoter architectures suited for different classes of recombinant proteins [32]. However, their predictive accuracy remains highly dependent on the availability of high-quality experimental datasets and therefore still requires iterative experimental validation.

Taken as a whole, promoter engineering in *K. phaffii* has evolved from empirical promoter optimization to systems-level transcriptional regulation [7,10]. Cis-regulatory and hybrid promoter engineering primarily optimize promoter architecture to fine-tune individual gene expression, whereas transcription factor engineering extends regulation to the network level through shared regulatory proteins. More recently, intelligent promoter design integrates endogenous stress responses, synthetic circuits, and data-driven modeling to achieve adaptive and context-dependent transcriptional regulation [31,32,36,50]. Rather than maximizing promoter strength alone, current strategies increasingly emphasize coordinated regulation of transcription, protein folding, secretion, and cellular metabolism, providing a foundation for predictive and self-adaptive expression systems in *K. phaffii*.

## 3. Signal Peptide Engineering: Mechanistic Determinants for Optimizing Protein Secretion

Efficient secretion of heterologous proteins represents one of the major advantages of the *K. phaffii* expression platform [2,4,8]. While promoter engineering determines [10] recombinant protein synthesis, successful extracellular production ultimately depends on efficient targeting of nascent polypeptides into the eukaryotic secretory pathway. Consequently, signal peptides have emerged as indispensable cis-acting elements that govern endoplasmic reticulum (ER) targeting, protein translocation, and the initiation of downstream folding and maturation processes [26,53]. Similar to other eukaryotic yeasts, the secretory pathway of *K. phaffii* is highly conserved with that of *S. cerevisiae*, allowing numerous signal peptide engineering strategies identified in the latter to be successfully translated into recombinant protein secretion in *K. phaffii* [16,54].

The effectiveness of signal peptide engineering is also influenced by the transcriptional control strategy used for recombinant protein expression. Under strong methanol-induced expression driven by PAOX1, secretion efficiency is often limited by transient overload of the ER folding machinery immediately after induction [8,25,36]. In contrast, constitutive and methanol-free expression systems typically experience lower instantaneous transcriptional stress but prolonged secretory demand [8,45]. Therefore, the optimal signal peptide and protein-targeting strategy may differ depending on the promoter system employed [17].

**Figure 4:**
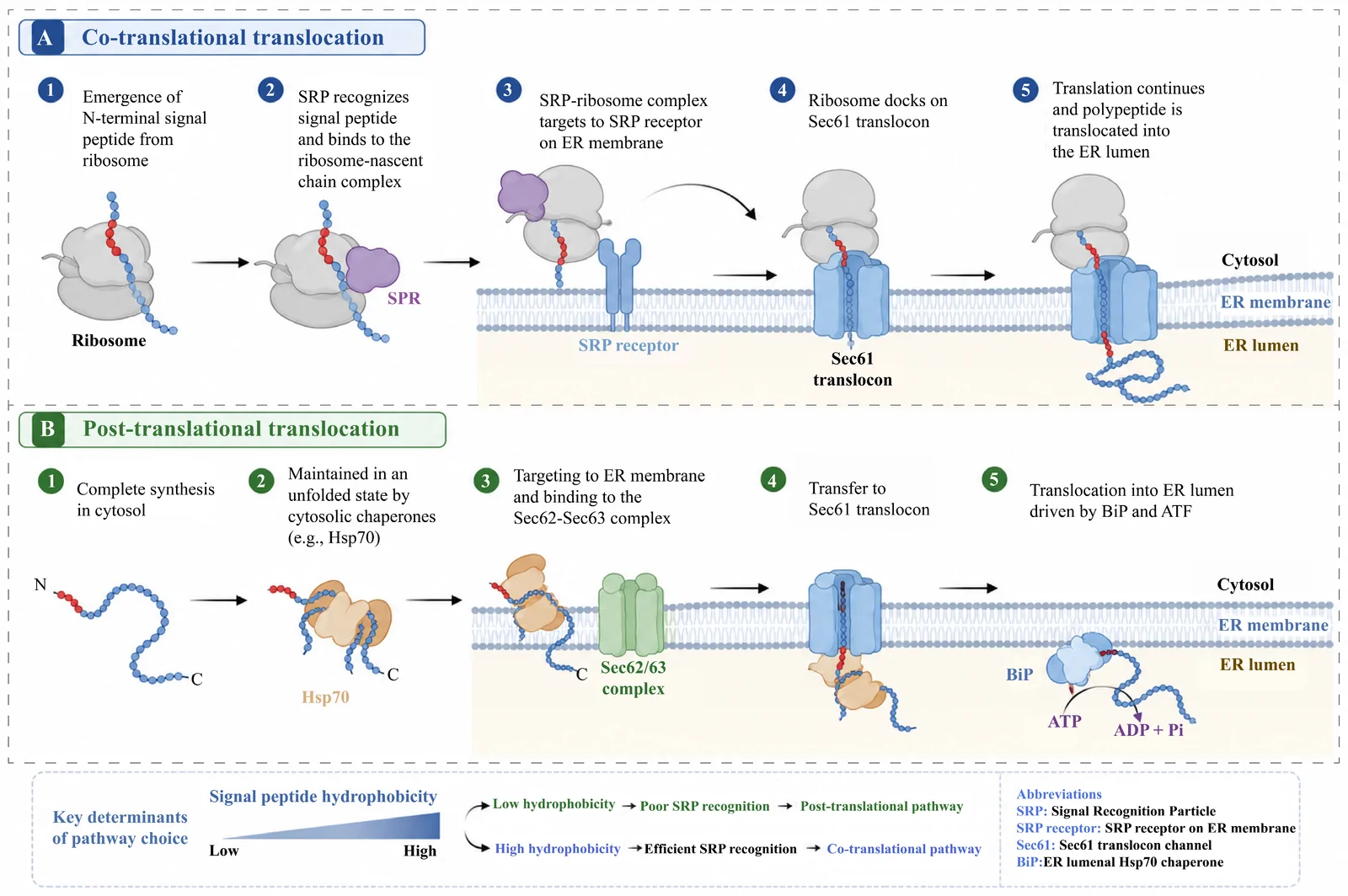
Structural organization of signal peptides and mechanisms of co-translational and post-translational protein translocation in *K. phaffii*.

Canonical signal sequences are short N-terminal peptides composed of three structurally and functionally distinct regions (Figure 4): a positively charged N-region that mediates the initial interaction with the ER membrane, a hydrophobic core (h-region) responsible for membrane insertion and signal recognition, and a C-region containing the signal peptidase cleavage site required for removal after ER entry [55,56,57]. Acting cooperatively, these domains regulate protein targeting, translocation efficiency, and early secretory processing, thereby establishing the molecular basis for post-translational modification, quality control, and extracellular secretion [57,58,59].

Recent studies have demonstrated that secretion signals influence multiple stages of the secretory pathway beyond ER targeting alone. Variations in sequence architecture affect signal recognition particle (SRP) recruitment, ER translocation kinetics, protein folding, intracellular trafficking, and ultimately recombinant protein yield [55,58,60]. Accordingly, protein translocation has become recognized as one of the principal bottlenecks limiting heterologous protein secretion in *K. phaffii* and other eukaryotic hosts [6,25,53]. These mechanistic insights have shifted signal peptide engineering from empirical signal replacement toward rational optimization of protein targeting, translocation efficiency, and secretory pathway utilization.

Protein targeting to the ER occurs through two mechanistically distinct routes—co-translational and post-translational translocation—which differ primarily in the timing of ER engagement (Figure 4) [55,59]. During co-translational translocation, the emerging signal sequence is recognized by the signal recognition particle (SRP) as it exits the ribosome. The resulting SRP–ribosome complex is subsequently delivered to the SRP receptor and the Sec61 translocon, coupling translation with ER import [23,58]. In contrast, post-translational translocation takes place after protein synthesis is completed in the cytosol. Cytosolic Hsp70 chaperones maintain precursor proteins in a translocation-competent state before delivering them to the Sec61–Sec62–Sec63 complex, where ATP-dependent cycles of BiP binding and release drive complete ER import [59].

Selection between these two pathways is largely determined by the physicochemical properties of the signal sequence, particularly the hydrophobicity of the h-region, which is a major determinant of SRP recognition [26,53]. Highly hydrophobic signals preferentially enter the co-translational route, whereas less hydrophobic sequences generally rely on post-translational transport. Because co-translational targeting introduces nascent polypeptides into the ER before extensive cytosolic folding occurs, it minimizes premature folding, aggregation, and translocon blockage, making this route particularly advantageous for large, multidomain, or structurally complex recombinant proteins [21,22].

These mechanistic insights have driven the evolution of rational engineering of signal-targeting pathwaysfrom empirical sequence replacement to rational engineering of protein-targeting pathways. Early studies focused on improving the classical *Saccharomyces cerevisiae* α-mating factor (α-MF) secretion signal, whereas subsequent work explored alternative co-translational signals and endogenous secretion sequences with superior compatibility to the native secretory machinery [22,26,61]. More recently, advances in synthetic biology and computational protein engineering have enabled the design of synthetic signal sequences and their integration with promoter architecture redesign, molecular chaperones, and ER quality-control pathways, extending enhancement of secretion efficiency toward systems-level regulation [25,53].

These mechanistic insights provide the molecular basis for rational engineering of signal peptides. Building upon these principles, diverse engineering strategies have been developed to improve ER targeting, protein translocation, and secretion efficiency in *K. phaffii*. The following sections summarize representative approaches, including rational engineering of the α-mating factor secretion signal, engineering of co-translational targeting sequences, and mining of endogenous signal peptides.

### 3.1. Rational Engineering of the α-Mating Factor Secretion Signal

The *Saccharomyces cerevisiae* α-mating factor (α-MF) secretion signal remains the most widely used leader sequence for heterologous protein secretion in *K. phaffii* because of its robust performance, broad substrate compatibility, and extensive experimental validation [21,26,53]. It has been successfully applied to the production of diverse recombinant proteins, including human collagen [22], β-galactosidase [61], and thaumatin [62]. Structurally, α-MF comprises an N-terminal Pre region responsible for endoplasmic reticulum (ER) targeting and a Pro region that promotes intracellular processing, protein folding, and Golgi trafficking before secretion [53,56]. This modular organization accounts for its broad applicability across different recombinant proteins.

Despite these advantages, the performance of α-MF is highly cargo-dependent. Secretion efficiency often declines for proteins with large molecular weights, multidomain architectures, or complex folding requirements [63]. This limitation primarily reflects its dependence on post-translational translocation, in which fully synthesized polypeptides are delivered to the Sec61 translocon after cytosolic folding has already begun [54,59,60]. Premature folding can impair translocation competence, increase intracellular retention, and reduce secretion efficiency. Furthermore, inefficient processing of the Pro region may promote protein aggregation within the ER, activate the unfolded protein response (UPR), and enhance ER-associated degradation (ERAD), further limiting recombinant protein production [53,59]. These observations demonstrate that secretion efficiency is determined not only by the secretion signal itself but also by its compatibility with protein folding and intracellular processing.

Mechanistic understanding of these limitations has guided successive optimization strategies. Early studies concentrated on sequence-level engineering through amino acid substitutions (for example, V50A), deletion of the third α-helix within the pro-region, and optimization of the Kex2 processing site significantly altered secretion efficiency. For example, Ito et al. [54] reported that the V50A substitution significantly enhanced secretion efficiency. Subsequent efforts adopted structure-guided redesign through insertions, deletions, and truncations of the Pro region. Freudl et al. [56] and Dalvie et al. [64] showed that reducing structural rigidity, particularly through deletion of the third α-helix, increased secretion by more than 50%, indicating that a more flexible conformation facilitates post-translational translocation through the Sec61 channel.

More recently, optimization has expanded beyond the secretion signal itself to include the signal peptide–cargo interface. Dalvie et al. [64] demonstrated that improving the N-terminal interface enhances recruitment of ER chaperones, thereby promoting protein folding and quality control. Likewise, Dai et al. [65] found that reducing steric hindrance around the Kex2 cleavage site improves Pro-region processing and accelerates secretion. Together, these studies indicate that efficient secretion depends on coordinated regulation of signal sequence architecture, cargo folding kinetics, and intracellular processing rather than on α-MF sequence engineering alone.

Recent studies demonstrate that α-MF sequence engineering has evolved from empirical sequence modification to mechanism-guided regulation of ER targeting, protein folding, and Golgi processing [26,53]. Nevertheless, because α-MF inherently relies on post-translational translocation, it remains suboptimal for many structurally complex recombinant proteins. These limitations have stimulated increasing interest in alternative secretion signals that exploit co-translational translocation, as discussed in the following section [22,26,53].

### 3.2. Rational Engineering of Co-Translational Signal Peptides

To overcome the intrinsic limitations of post-translational translocation, recent studies have increasingly explored co-translational targeting sequences that utilize the signal recognition particle (SRP)-dependent pathway [26,53,55]. Compared with the classical α-MF secretion signal, these sequences possess more hydrophobic h-regions, promoting rapid SRP recognition and directing nascent polypeptides to the endoplasmic reticulum (ER) membrane during translation [26,55].

Among the currently available secretion signals, the Ost1-derived leader sequence has become the best-characterized co-translational targeting module. Its highly hydrophobic core facilitates efficient SRP recruitment immediately after emergence from the ribosome, thereby coupling protein synthesis with ER import and minimizing cytosolic residence of nascent chains [26,59,66]. This mechanism reduces premature folding, protein aggregation, and translocon blockage, making it particularly advantageous for recombinant proteins with large molecular weights, multidomain architectures, or complex folding requirements [21,59,66].

Building on this mechanism, a widely adopted strategy replaces the 19-amino-acid α-MF pre-region with the highly hydrophobic OST1 signal peptide while retaining the α-MF pro-region. This chimeric design combines efficient co-translational ER targeting with the well-established Golgi processing efficiency of α-MF, thereby integrating the advantages of both secretion systems [59,66].

The effectiveness of this strategy has been demonstrated across multiple recombinant proteins. Barrero et al. [23], Wang et al. [67], and Zhang et al. [66] independently reported substantial improvements in secretion efficiency using Ost1-based hybrid leaders. In particular, Barrero et al. achieved a 10–20-fold increase in recombinant protein secretion [23], whereas Zhang et al. observed enhanced glycosaminoglycan synthase expression accompanied by an approximately 20% increase in hyaluronic acid production [66]. These results demonstrate that optimizing the ER-targeting route can, for some proteins, produce greater improvements than sequence modification alone.

Despite these advantages, co-translational targeting is not universally optimal. Its performance remains highly dependent on cargo-specific characteristics, including folding kinetics, structural complexity, and post-translational modification requirements [26,53]. Consequently, α-MF- and Ost1-based secretion systems should be regarded as complementary platforms rather than universally interchangeable solutions.

Viewed together, engineering of secretion signal architecture has evolved from empirical sequence engineering toward mechanistic regulation of ER-targeting pathways [26,53]. Nevertheless, the persistent cargo dependence of both post-translational and co-translational systems has prompted increasing interest in endogenous secretion signals that are naturally adapted to the *K. phaffii* secretory machinery, as discussed in the following section [53].

### 3.3. Mining Endogenous Signal Peptides: Host Compatibility and Cargo-Specific Compatibility

Although engineering of α-MF and other heterologous secretion signals has substantially improved recombinant protein production, their performance remains highly cargo-dependent [26,53]. This limitation has stimulated increasing interest in endogenous secretion signals derived from the *K. phaffii* genome, which have co-evolved with the native secretory machinery and therefore exhibit enhanced compatibility with endoplasmic reticulum (ER) targeting, protein translocation, and intracellular processing [26,53,68].

Genome-wide screening has identified numerous endogenous secretion signals, including Dan4, Gas1, Msb2, Fre2 [68], SP23 [69], and many additional candidates discovered through systems biology and comparative genomics [53]. Because these signals originate from naturally secreted proteins, they generally display improved coordination with signal recognition particle (SRP) recruitment, Sec61-mediated translocation, and downstream folding and trafficking processes [26,53].Representative endogenous signal peptides and their reported applications in heterologous protein secretion in *K. phaffii* are summarized in Table 2.

Advances in multi-omics analysis and high-throughput screening have further accelerated the discovery of efficient secretion signals. Duan et al. [68] identified several high-performance endogenous leaders, among which Msb2 increased secretion of enhanced green fluorescent protein (EGFP) and multiple recombinant proteins by 1.5–8-fold relative to α-MF. Likewise, Liang et al. [70] systematically evaluated Scw, Dse, and Exg signal sequences and demonstrated that most outperformed α-MF, although their advantages remained strongly dependent on the recombinant cargo.

These studies indicate that secretion performance cannot be explained by any single structural feature. Instead, it arises from the combined effects of signal sequence length, N-region charge, hydrophobic core composition, and cleavage-site architecture, which together influence SRP recognition, translocon engagement, and subsequent protein maturation [69].

Despite their superior compatibility with the native secretion machinery, endogenous signals also exhibit pronounced cargo specificity. Shen et al. [71] evaluated the secretion potential of the PAS_chr3_0030 signal peptide derived from *K. phaffii* and demonstrated that it supported the secretion of approximately 75% of the tested enzymes. However, its performance varied substantially among different protein substrates. These results indicate that no endogenous secretion signal is universally optimal, even within its native host, highlighting the necessity of identifying cargo-compatible secretion elements.

To address this challenge, two complementary strategies have emerged. One strategy focuses on protein-specific screening to identify the most suitable endogenous signal peptide for individual recombinant proteins, whereas the other aims to establish diverse signal peptide repertoires through large-scale comparative screening. Using comparative analyses of secretion signals from different yeast species, Zou et al. [53] identified several highly effective endogenous leaders, including chr3_0517, chr1-4_0584, chr2-1_0140, and chr3_0960, thereby expanding the available repertoire of secretion elements for heterologous protein production. These findings demonstrate that broad exploration of endogenous signal peptide diversity provides valuable resources for overcoming the limitations of single-signal approaches and enables more rational matching between signal peptides and recombinant cargoes.

Bioinformatic analyses further demonstrate the remarkable structural diversity of endogenous secretion signals. Massahi et al. [69] revealed extensive variation in sequence length, charge distribution, hydrophobicity, and amino acid composition across the *K. phaffii* secretome. Among the candidates evaluated, SP23 exhibited secretion efficiency comparable to that of α-MF under industrial fermentation conditions, highlighting its potential as an alternative secretion leader [69].

These advances have shifted the field from reliance on a single universal leader toward the development of diverse, cargo-adapted secretion toolkits [26,53]. Nevertheless, accurately predicting optimal signal sequence–cargo combinations remains a major challenge because the molecular determinants governing their compatibility are still incompletely understood. Integrating high-throughput screening with computational prediction and machine learning is expected to accelerate the rational selection and design of next-generation secretion signals [25].

**Table 2:** Representative endogenous signal peptides evaluated for heterologous protein secretion in *K. phaffii*.

Signal Peptide	Category	Source Protein	Target Recombinant Protein	Relative Secretion Efficiency (α-MF Used as the Reference Control)	Reference
Dan4	Cell wall protein	Cell wall protein Dan4	Cephalosporin C acylase	3.8-fold increase	Duan et al., 2019 [68]
Gas1	Secreted enzyme	β-1,3-glucanosyltransferase	β-Galactosidase	Significant increase	Duan et al., 2019 [68]
Msb2	Membrane protein	Mucin-like signaling protein	EGFP	1.5-fold increase	Duan et al., 2019 [68]
Fre2	Membrane protein	Ferric/cupric reductase	EGFP	1.4-fold increase	Duan et al., 2019 [68]
Dse4	Cell wall protein	Daughter cell separation protein	EGFP/CalB	Comparable to α-MF	Liang et al., 2012 [70]
Exg1	Secreted enzyme	Exo-β-1,3-glucanase	EGFP/CalB	Comparable to α-MF	Liang et al., 2012 [70]
Ost1	ER membrane protein	Oligosaccharyltransferase subunit Ost1	BTL2 lipase	10–20-fold increase	Barrero et al., 2018 [23]
SP23	Predicted signal peptide	Predicted secreted protein	rhGH	Comparable to α-MF	Massahi et al., 2016 [69]
PAS_chr3_0030	Genome-mined signal peptide	Predicted secreted protein	Multiple enzymes	Conferred secretion competence (α-MF failed to secrete this enzyme)	Shen et al., 2022 [71]

Note: In the original studies, endogenous signal peptides were evaluated by replacing the conventional α-mating factor (α-MF) secretion signal while keeping all other experimental conditions unchanged. Therefore, secretion performance is presented relative to the α-MF control, which served as the standard benchmark for assessing signal peptide efficiency. Absolute recombinant protein titers (g L^−1^ or mg L^−1^) were generally not reported because these studies primarily focused on comparative evaluation of signal peptide performance rather than process-scale protein production.

## 4. Molecular Chaperones and Folding Cofactors: Enhancing Proper Protein Folding Efficiency

Although promoter engineering and signal peptide optimization effectively increase recombinant protein synthesis and facilitate endoplasmic reticulum (ER) targeting, efficient extracellular production ultimately relies on the capacity of the host secretory machinery to correctly fold, process, and transport newly synthesized proteins [25,26,53] as recombinant protein synthesis increases the ER [24,72]. This stress activates the unfolded protein response (UPR), which expands the protein-folding machinery, together with the endoplasmic reticulum-associated degradation (ERAD) pathway, which removes irreversibly misfolded proteins [6,24]. Although these adaptive responses transiently restore proteostasis, persistent ER stress ultimately limits recombinant protein secretion and remains a major bottleneck in high-level heterologous protein production in *K. phaffii* [6,24,72].

Similarly, the benefits of molecular chaperone co-expression depend strongly on the transcriptional background. In PAOX1-driven production, chaperones mainly alleviate acute ER stress triggered by high transcriptional bursts after methanol induction [17,24,36]. Conversely, under constitutive promoters such as PGAP or PTEF1, chaperone engineering primarily supports long-term protein-folding homeostasis during continuous recombinant protein synthesis [8,73].

To overcome these limitations, engineering strategies have progressively shifted from simply increasing protein synthesis toward enhancing the processing capacity of the secretory machinery [24,25]. Current approaches primarily focus on overexpression or combinatorial co-expression of ER-resident molecular chaperones, protein-folding catalysts, and UPR regulators to improve protein maturation while maintaining intracellular proteostasis [6,23,24,25]. These folding components, including Kar2/BiP, PDI1, Ero1, Hac1, and the Ire1-mediated UPR signaling pathway, are highly conserved between *S. cerevisiae* and *K. phaffii*, which explains why many molecular chaperone engineering strategies were initially developed in *S. cerevisiae* before being adapted to *K. phaffii*. Nevertheless, quantitative optimization remains necessary because the two yeasts differ in transcriptional regulation, methanol metabolism, and industrial fermentation physiology. Representative targets include the molecular chaperone BiP/Kar2p, protein disulfide isomerase (*PDI1*), the oxidoreductase Ero1p, and the master UPR regulator Hac1P [6,24]. Rather than functioning as independent engineering modules, these regulators are increasingly combined with upstream regulatory and targeting strategies to improve protein maturation and secretion efficiency [25].

**Figure 5:**
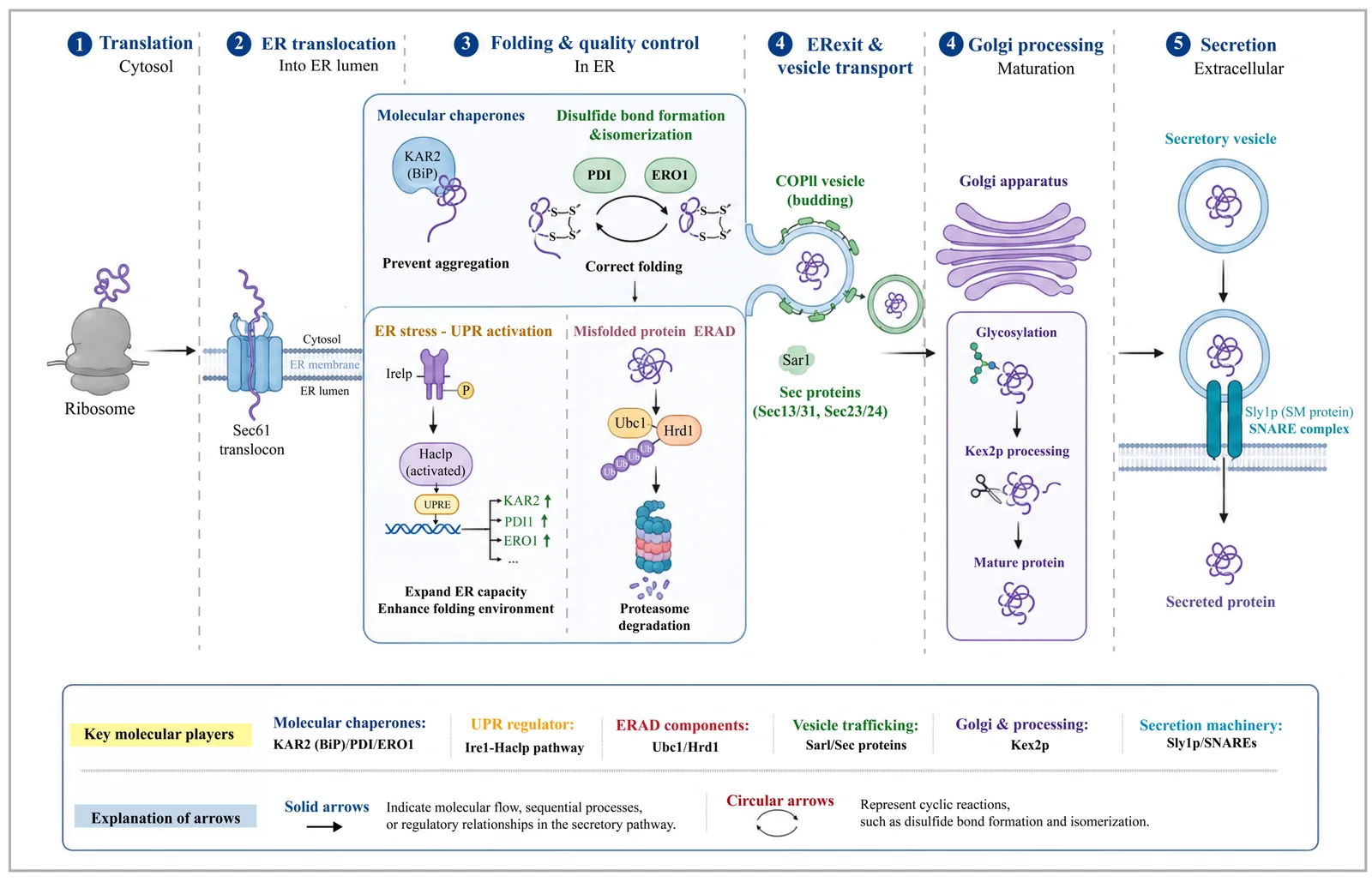
Schematic overview of engineering targets along the secretory pathway in *K. phaffii*. Recombinant proteins enter the ER for folding and quality control before trafficking through the Golgi apparatus for secretion. Molecular chaperones, protein-folding catalysts, the UPR, ER-associated degradation (ERAD), vesicle trafficking, and translational regulation collectively determine secretion efficiency and therefore represent major targets for secretory pathway engineering.

As illustrated in Figure 5, recombinant protein secretion involves coordinated protein folding, quality control, vesicle trafficking, and extracellular transport, providing multiple opportunities for engineering intervention. Accordingly, current strategies have evolved from overexpression of individual folding factors to systems-level remodeling of the entire secretory pathway, establishing an important framework for constructing high-performance *K. phaffii* cell factories [66,69].

### 4.1. ER-Resident Molecular Chaperones and Protein Folding Enzymes

ER-resident molecular chaperones and folding catalysts constitute the core quality-control machinery responsible for maintaining intracellular proteostasis by facilitating nascent polypeptide folding, catalyzing disulfide bond formation, and preventing protein aggregation [23,24,25]. The major ER-resident molecular chaperones and UPR signaling components, including Kar2/BiP, Pdi1, Ero1, Ire1, and Hac1, are highly conserved between *S. cerevisiae* and *K. phaffii*. Consequently, many molecular chaperone engineering and UPR regulation strategies were first established in *S. cerevisiae* and subsequently adapted to improve recombinant protein secretion in *K. phaffii* [14,17,25]. Among the best-characterized components are the Hsp70-family chaperone BiP (Kar2p), which stabilizes unfolded proteins, protein disulfide isomerase (*PDI1*), which catalyzes disulfide bond formation and isomerization, and the oxidoreductase Ero1p, which regenerates oxidized *PDI1* to sustain an appropriate oxidative environment for protein maturation [6,23,24]. As recombinant protein production has increased, engineering strategies have progressively shifted from single-gene overexpression to combinatorial co-expression of ER folding factors. Accordingly, current approaches can be broadly classified into three categories: single-factor enhancement, combinatorial chaperone engineering, and systems-level expansion of the ER folding network.

#### 4.1.1. Single-Chaperone Enhancement

Enhancing individual folding catalysts is particularly effective for recombinant proteins containing multiple disulfide bonds or complex tertiary structures, where specific folding reactions become rate limiting [24]. Among the available targets, *PDI1* and the peptidyl-prolyl cis–trans isomerase *CPR5* have demonstrated particularly strong effects because they accelerate disulfide bond maturation and proline isomerization, respectively. During heterologous expression of carboxylesterase FumDM, co-expression of *PDI1* and *CPR5* increased enzyme activity by 635% and 357%, respectively, substantially outperforming overexpression of *ERO1*, *HAC1*, and BiP [74]. These results demonstrate that identifying and relieving the dominant folding bottleneck is often more effective than indiscriminate overexpression of multiple folding factors.

#### 4.1.2. Combinatorial Chaperone Co-Expression

Because protein maturation requires coordinated folding, disulfide bond formation, and quality control, simultaneous engineering of complementary folding factors generally provides greater benefits than individual chaperone overexpression [24,25]. In particular, combining molecular chaperones that stabilize nascent polypeptides with enzymes responsible for oxidative folding effectively alleviates multiple kinetic constraints during protein maturation. A representative example is the coordinated expression of *KAR2*, *PDI1*, and *HAC1* in a nanobody production platform, where *PDI1* and *KAR2* increased antibody secretion by approximately 14-fold and 5-fold, respectively [73]. These results demonstrate that synergistic interactions among multiple ER-resident folding factors substantially improve the production of structurally complex recombinant proteins.

#### 4.1.3. Global Expansion of the ER Folding Network

As recombinant protein production reaches industrially relevant levels, the optimization of individual folding reactions alone is frequently insufficient to sustain efficient secretion [25]. Recent advances have shifted the focus toward systems-level remodeling of the ER folding network by coordinating protein translocation, folding, and quality-control pathways. Representative studies have shown that integrating promoter optimization with co-expression of multiple ER-resident folding factors markedly alleviates ER stress and improves secretion of structurally complex proteins such as human type III collagen, achieving titers of 10.3 g L^−1^ in a 5-L fermenter [21]. Likewise, coordinated activation of endogenous folding components, including Kar2p, Pdi1p, and Sec63, significantly enhanced stem bromelain production under constitutive expression conditions, further demonstrating that expansion of the endogenous folding network strengthens intracellular quality control during sustained high-level protein production [75]. These findings indicate that coordinated remodeling of the ER folding environment generally provides greater benefits than manipulation of individual folding catalysts alone.

These advances have transformed engineering of ER-resident folding factors from enhancing individual chaperones to systematic remodeling of the ER folding network [24,25]. Nevertheless, the effectiveness of individual folding factors remains highly protein dependent, varying with molecular size, structural complexity, disulfide bond content, and folding kinetics. Future strategies are therefore expected to integrate protein-specific folding modules, ER quality-control mechanisms, and secretory pathway engineering to establish more efficient and robust secretion platforms. As the principal regulator coordinating these processes, the unfolded protein response (UPR) has become a major target for quantitative engineering and is discussed in the following section [6,24].

### 4.2. Fine-Tuned Regulation of UPR Transcriptional Regulators

Whereas ER-resident molecular chaperones primarily relieve specific protein-folding bottlenecks, activation of the unfolded protein response (UPR) provides a global adaptive mechanism that maintains endoplasmic reticulum (ER) proteostasis during high-level recombinant protein production. In *K. phaffii*, the master UPR transcription factor Hac1P is generated through Ire1-mediated unconventional splicing of *HAC1* mRNA in response to ER stress. Activated Hac1P subsequently induces a broad transcriptional program that enhances protein folding, disulfide bond formation, glycosylation, vesicle trafficking, and ER-associated degradation (ERAD), thereby expanding the processing capacity of the secretory pathway and restoring intracellular protein homeostasis [6,24].

Despite these advantages, increasing UPR activity does not necessarily result in higher recombinant protein yields. Although constitutive *HAC1* overexpression can enhance the ER folding machinery, excessive activation frequently imposes a substantial metabolic burden, impairs cell growth, and may disrupt intracellular homeostasis [6,24]. These limitations have prompted recent engineering strategies to shift from constitutive activation toward quantitative modulation of UPR activity to achieve a balance between recombinant protein synthesis and the processing capacity of the secretory pathway [24,25]. This transition has established the foundation for integrating UPR regulation with promoter architecture redesign, molecular chaperones, translational control, and co-translational signal peptide engineering to improve recombinant protein secretion at the systems level.

#### 4.2.1. Fine-Tuning of HAC1 Expression

Precise modulation of *HAC1* expression has emerged as an effective strategy for optimizing UPR activity [6,24,25]. Rather than maximizing transcription, moderate UPR activation generally provides superior recombinant protein production by expanding ER folding capacity while avoiding excessive metabolic burden [6,24]. A representative study demonstrated that replacing the native *HAC1* promoter with the weaker P55 promoter achieved optimal UPR activity, increasing nanobody secretion by 2.41-fold and yielding a final titer of 2.13 g L^−1^ without compromising cell growth [76]. These results demonstrate that quantitative regulation, rather than constitutive overexpression, is essential for balancing secretory capacity with cellular fitness. Collectively, current studies highlight a transition from empirical *HAC1* overexpression toward precise tuning of UPR activity to maximize recombinant protein secretion [24].

#### 4.2.2. Systems-Level Coordination of the Secretory Pathway

Beyond quantitative regulation of *HAC1*, recent engineering strategies increasingly integrate UPR modulation with complementary components of the secretory pathway to coordinate protein synthesis, ER translocation, folding, and secretion [24,25]. This systems-level approach alleviates bottlenecks throughout the secretory process rather than improving individual regulatory modules in isolation.

Representative studies demonstrate the effectiveness of this strategy. Integration of promoter fine-tuning with co-expression of ER-resident folding factors markedly alleviated ER stress during recombinant human type III collagen production, achieving a fermentation titer of 10.3 g L^−1^ in a 5-L fermenter [21]. Likewise, coordinated expression of *HAC1*, *PDI1*, and the translation initiation factor Pab1 improved the balance between protein synthesis and folding, increasing xylanase production to 11.8 g L^−1^ [77]. In addition, coupling co-translational signal peptide engineering with *HAC1* regulation synchronized ER translocation with protein folding, resulting in a 4.9-fold increase in recombinant human hyaluronidase secretion [66]. Collectively, these studies demonstrate that integrating UPR regulation with promoter engineering, molecular chaperones, translational control, and signal peptide optimization generally provides greater improvements than manipulating *HAC1* alone [21,66,77]. Taken together, current studies suggest that UPR engineering has progressed from constitutive activation of individual regulators to quantitative and systems-level regulation of ER homeostasis. Future studies are expected to integrate dynamic UPR regulation with promoter architecture redesign, translational control, ER translocation, molecular chaperones, and vesicle trafficking to maximize recombinant protein secretion while minimizing metabolic burden. Representative applications of molecular chaperone co-expression and UPR regulation in *K. phaffii* are summarized in Table 3.

## 5. Systems-Level Coordination and Dynamic Regulatory Strategies

Efficient heterologous protein secretion in *K. phaffii* depends on the coordinated regulation of multiple interconnected processes, including transcription, endoplasmic reticulum (ER) translocation, protein folding, post-translational modification, intracellular trafficking, and quality control [6,23,24,25]. Consequently, improving secretion efficiency requires coordinated optimization of the entire secretory pathway rather than independent enhancement of individual expression modules. Although engineering of promoters, signal peptides, and molecular chaperones has substantially increased recombinant protein production, further improvements are increasingly constrained by imbalances among these cellular processes [24,25]. Simply combining multiple high-performance expression elements frequently perturbs intracellular homeostasis, resulting in excessive ER stress, inefficient protein trafficking, and increased metabolic burden. These limitations have shifted current engineering strategies from optimizing individual components toward systems-level coordination of the secretory network.

High-throughput combinatorial engineering has emerged as an effective approach for simultaneously optimizing multiple regulatory modules. Libraries integrating promoter variants, signal peptides, molecular chaperones, and other expression elements enable rapid identification of combinations that improve protein synthesis, ER translocation, and folding while maintaining balanced intracellular resource allocation [78]. However, the performance of these optimized combinations is often highly context dependent. During industrial fermentation, fluctuations in substrate availability, oxygen transfer, and other environmental parameters alter cellular physiology, frequently disrupting the regulatory balance established under laboratory conditions. These observations highlight that static optimization alone is insufficient to accommodate the dynamic physiological changes encountered during large-scale bioprocesses.

To address these limitations, increasing attention has been directed toward dynamic regulatory systems capable of continuously adapting recombinant protein production to the physiological state of the host cell [79,80]. Synthetic gene circuits represent a particularly promising strategy because they exploit endogenous regulatory signals rather than relying exclusively on external induction. Representative examples include UPR-responsive promoters, such as *PKAR2*, and synthetic promoters containing unfolded protein response elements (UPREs), which activate molecular chaperones and other folding-associated factors only under conditions of elevated ER stress and gradually return to basal activity once proteostasis has been restored [79,80]. Compared with constitutive expression, these feedback-regulated systems reduce unnecessary metabolic expenditure while maintaining efficient protein folding and secretion.

Dynamic regulation can also be achieved through temporal coordination of gene expression during different stages of cell growth. Growth phase-dependent promoters preferentially allocate metabolic resources to biomass accumulation during exponential growth before progressively redirecting cellular resources toward recombinant protein production during high-cell-density fermentation [81]. By dynamically adjusting transcriptional activity according to cellular growth and stress states, these temporally regulated systems improve resource allocation and support efficient production of structurally complex proteins [23,81]. Together with stress-responsive regulatory circuits, these strategies illustrate the advantages of adaptive regulation over static engineering for balancing host physiology with recombinant protein production.

Recent studies demonstrate that heterologous protein production in *K. phaffii* is shifting from static optimization of individual components toward adaptive regulation of host physiology and production performance [6,24,25]. Future progress will increasingly depend on programmable regulatory systems that couple recombinant protein production with cellular physiological states. In this context, synthetic biology provides programmable regulatory modules, whereas AI-assisted design is expected to improve the selection of compatible regulatory combinations from large design spaces rather than relying solely on empirical screening [32,78]. Such integration is particularly valuable for optimizing multi-factor engineering strategies whose experimental search space would otherwise become prohibitively large [78,79]. Nevertheless, the robustness, scalability, and long-term stability of these regulatory systems under industrial fermentation conditions remain insufficiently understood. Overcoming these challenges will be critical for establishing next-generation *K. phaffii* cell factories capable of robust, efficient, and sustainable production of structurally complex recombinant proteins.

## 6. Conclusions and Future Perspectives

Over the past decade, the development of *K. phaffii* expression technologies has progressed from isolated engineering of individual genetic elements toward more comprehensive optimization of host cellular functions and production processes. Advances in transcriptional regulation, protein targeting, intracellular folding, and adaptive control have collectively expanded the production capacity of this yeast, enabling more efficient synthesis of diverse recombinant products [6,24,27,30,59]. Nevertheless, manufacturing structurally complex biomolecules—including multimeric proteins, highly glycosylated proteins, and disulfide-rich proteins—remains constrained by the intricate interplay among biosynthesis, intracellular processing, and cellular homeostasis [6,23,24,25]. Moreover, different classes of recombinant proteins, including soluble enzymes, membrane proteins, multimeric complexes, and proteins requiring extensive post-translational processing, impose distinct physiological demands on the host cell and therefore require different engineering strategies [24,25]. These challenges indicate that improving isolated regulatory modules alone is unlikely to eliminate the remaining limitations of recombinant protein production.

Beyond academic and pilot-scale applications, the successful commercialization of *K. phaffii*-derived recombinant proteins further demonstrates the industrial maturity and practical applicability of this platform [4,8]. At the same time, commercially relevant production requires not only high expression levels but also consistent product quality, genetic stability, and scalable fermentation performance [6,8]. Therefore, future engineering strategies should focus on establishing predictable and robust cell factories capable of meeting industrial manufacturing requirements [7].

Future progress will increasingly depend on integrated, protein-specific strategies that coordinate multiple cellular processes rather than simply enhancing individual expression modules. One promising direction is the development of methanol-independent production platforms coupled with adaptive regulatory circuits that continuously respond to endogenous physiological signals. Such self-regulating systems are expected to coordinate cellular metabolism with biosynthetic demand, reducing reliance on external induction while improving process safety, sustainability, and industrial compatibility [6,24,51,80].

Meanwhile, advances in high-throughput design and automated screening are reshaping the development of customized production platforms. Combinatorial libraries incorporating diverse regulatory elements, together with rapid screening technologies, will facilitate identification of optimal configurations for proteins with distinct structural and functional requirements [27,30,53,68]. As a result, future platform development is likely to shift from generalized engineering strategies toward protein-specific design, enabling rational selection of regulatory architectures according to the characteristics of individual recombinant products.

Artificial intelligence and machine learning are also expected to become important supporting tools for next-generation strain engineering rather than standalone solutions. Their primary value lies in integrating large-scale regulatory sequence datasets, multi-omics information, and experimental measurements to prioritize engineering targets, predict promoter activities, recommend signal peptide–cargo combinations, and guide the design of synthetic regulatory circuits before laboratory validation [31,32,35]. These capabilities are expected to reduce the experimental burden associated with combinatorial engineering, particularly when optimizing multiple regulatory elements simultaneously [27,78]. However, the predictive performance of current models remains constrained by the availability and quality of experimentally validated datasets, meaning that iterative experimental verification will continue to be indispensable for practical strain development [32,35].

Despite these advances, several challenges remain before predictive cell-factory design can be fully realized. Dynamic regulatory systems must demonstrate greater robustness, genetic stability, and scalability under industrial fermentation conditions, whereas AI-assisted engineering continues to depend on comprehensive, high-quality datasets for reliable model training and validation. Moreover, the molecular interactions linking gene regulation, protein biogenesis, intracellular trafficking, and metabolic adaptation remain incompletely understood, limiting accurate prediction of cellular behavior during large-scale recombinant protein production [6,24,25].

Ultimately, the next generation of *K. phaffii* cell factories will be defined by predictive design and adaptive regulation, enabling more reliable and efficient strain development beyond conventional trial-and-error approaches. Achieving this vision will require continued integration of synthetic biology, systems metabolic engineering, artificial intelligence, and high-throughput experimentation. Rather than replacing experimental engineering, these approaches can facilitate hypothesis-driven design and reduce the experimental burden associated with strain development [6,24,32,35,51]. Many of the engineering strategies discussed in this review have benefited from the extensive mechanistic knowledge established in the genetically well-characterized yeast *Saccharomyces cerevisiae*. Continued advances in CRISPR-based genome editing, systems biology, and multi-omics technologies are expected to further accelerate the translation of these mechanistic insights into *K. phaffii*, enabling more rational engineering of promoters, signal peptides, molecular chaperones, and other expression elements [14,15,16,42]. Such advances are expected to accelerate the sustainable manufacture of biopharmaceuticals, industrial enzymes, food proteins, and other structurally complex, high-value recombinant proteins, further consolidating *K. phaffii* as a leading eukaryotic microbial cell factory for industrial biotechnology.

## Figures and Tables

**Table 3:** Representative engineering strategies targeting molecular chaperones and the unfolded protein response (UPR) to enhance heterologous protein secretion in *K. phaffii*.

Engineering Strategy	Target Protein	Engineering Target	Representative Approach	Evaluation Metric (Reported in the Original Study)	Reference
Single-chaperone enhancement	FumDM	*PDI1*/*CPR5*	Methanol-inducible co-expression	Enzyme activity increased by 635% (PDI1) and 357% (CPR5)	Jiang et al., 2023 [74]
Multi-chaperone co-expression	Bivalent nanobody	*KAR2* + *PDI1* + *HAC1*	Universal expression platform	Nanobody production increased by up to 15-fold	De Groeve et al., 2023 [73]
Endogenous ER network activation	Stem bromelain	*HAC1*-regulated endogenous network	Constitutive PGAP expression	Stem bromelain activity reached 54 U mL^−1^	Varadharaj et al., 2025 [75]
*HAC1* fine-tuning	Nanobody	*HAC1*	P55 promoter fine-tuning	Nanobody production increased 2.41-fold, reaching 2.13 g L^−1^	Zheng et al., 2024 [76]
ER folding network expansion	hlCOLIII α1	ER-resident chaperones	Promoter tuning	Collagen production reached 10.3 g L^−1^	Wang et al., 2025 [21]
Translation-folding coordination	Xylanase	*HAC1* + *PDI1* + Pab1	High-copy PAOX1 expression	Xylanase production reached 11.8 g L^−1^	Liu et al., 2024 [77]
ER translocation-UPR coupling	rhPH-20	Co-translational SP + *HAC1*	Signal peptide-UPR coupling	Hyaluronidase secretion increased 4.9-fold	Zhang et al., 2024 [66]

Note: Studies are grouped according to their primary engineering strategy, including single-factor enhancement, quantitative UPR regulation, combinatorial chaperone engineering, ER folding network remodeling, and systems-level coordination of protein synthesis, folding, and secretion. Abbreviations: UPR, unfolded protein response; ER, endoplasmic reticulum. The evaluation metrics shown in this table follow those reported in the original publications. Depending on the target protein and study objectives, recombinant protein production was assessed using volumetric protein titer (g L^−1^), enzymatic activity (U mL^−1^), or relative fold improvement. Therefore, these values should be interpreted within the context of the corresponding experimental system rather than being directly compared across studies.

## Data Availability

No new data were created or analyzed in this study. Data sharing is not applicable to this article.
